# Oral infection with periodontal pathogens induced chronic obstructive pulmonary disease-like lung changes in mice

**DOI:** 10.1186/s12903-024-04635-6

**Published:** 2024-07-26

**Authors:** Wenyue Li, Wenyan Liu, Hongjia Yang, Xueyuan Wang, Zuomin Wang, Zhiqiang Liu

**Affiliations:** 1 Department of Stomatology, Beijing Chao-Yang Hospital, Capital Medical University, 8 Gongti South Road, Chaoyang District, Beijing, 100020 China; 2 Department of Stomatology, Beijing Lu He Hospital, Capital Medical University, Beijing, China

**Keywords:** Periodontitis, Chronic obstructive pulmonary disease, Inflammation, *Porphyromonas gingivalis*, *Fusobacterium nucleatum*

## Abstract

**Background:**

Epidemiological studies have demonstrated that periodontitis is an independent risk factor for chronic obstructive pulmonary disease (COPD). However, the mechanism underlying the association between these two diseases remains unclear. The lung microbiota shares similarities with the oral microbiota, and there is growing evidence to suggest that the lung microbiome could play a role in the pathogenesis of COPD. This study aimed to investigate whether periodontal pathogens could contribute to the pathogenesis of COPD in a mouse model.

**Methods:**

We established mouse models with oral infection by typical periodontal pathogens, *porphyromonas gingivalis* (Pg group) or *fusobacterium nucleatum* (Fn group), over a three-month period. Mice that did not receive oral infection were set as the control group (C group). We assessed the level of alveolar bone resorption, lung function, and histological changes in the lungs of the mice. Additionally, we measured the levels of inflammatory factors and tissue damage associated factors in the lung tissues.

**Results:**

Lung function indices, including airway resistance, peak inspiratory/expiratory flow and expiratory flow-50%, were significantly reduced in the Fn group compared to the C group. Additionally, histological examination revealed an increased number of inflammatory cells and bullae formation in the lung tissue sections of the Fn group. Meanwhile, levels of inflammatory factors such as IL-1β, IL-6, IFN-γ, and TNF-α, as well as tissue damage associated factors like matrix metalloproteinase-8 and neutrophil elastase, were significantly elevated in the lung tissue of the Fn group in comparison to the C group. The Pg group also showed similar but milder lung changes compared to the Fn group. Pg or Fn could be detected in the lungs of both oral infected groups.

**Conclusion:**

The results indicated that oral periodontal pathogens infection could induce COPD-like lung changes in mice, and they may play a biological role in the association between periodontitis and COPD.

## Introduction

Chronic obstructive pulmonary disease (COPD) is a highly prevalent chronic respiratory disease and characterized by irreversible airflow obstruction. It has become the third leading cause of death worldwide, and the prevalence of COPD is expected to increase with the aging of the global population [1]. The pathogenesis of COPD is not fully understood. Although tobacco smoking is considered the primary risk factor, emerging evidence suggests that other risk factors are also significant [2].

In recent years, increased epidemiological evidence has demonstrated that periodontitis is an independent risk factor for COPD [3]. As one of the most common oral diseases, severe periodontitis affects approximately 10.8% of the global population [4]. It primarily presents as chronic inflammation of the periodontal tissues and alveolar bone resorption, contributing to one of the leading causes of tooth loss. Infection with periodontal pathogens is recognized as the initiating cause of periodontitis [5]. Additionally, an intervention study has shown that periodontal therapy in COPD patients with periodontitis can improve lung function and reduce the frequency of COPD exacerbations [6]. Despite this, the underlying mechanism linking the two diseases is not yet fully understood.

Recently, growing evidence has shown that the lung microbiome may contribute to COPD pathogenesis, and it altered during the course of the disease [7]. The lung microbiota exhibits a higher degree of similarity to the oral microbiota than to the nasal microbiota in both healthy individuals and those with COPD, suggesting that oral bacteria may be an important source of lung bacteria in both groups [8, 9]. However, the role of oral bacteria in the pathogenesis of COPD is not yet well understood. Studies have found that periodontal pathogens can be detected in respiratory secretions of COPD patients [10]. Furthermore, periodontal pathogens have been demonstrated to play significant roles in the association between periodontitis and various systemic diseases, including cardiovascular disease, Alzheimer’s disease, and others [11, 12].

Therefore, we hypothesize that periodontal pathogens from the oral cavity of individuals with periodontitis may migrate to the lungs and contribute to the pathogenesis of COPD. In this study, we established mouse models of oral infection with typical periodontal pathogens, *Porphyromonas gingivalis* (*P. gingivalis*) and *Fusobacterium nucleatum* (*F. nucleatum*), and determined the changes in lung tissue and lung function to identify the potential effects of these pathogens on COPD pathogenesis.

## Materials and methods

### Mice

Thirty-six 7–8 weeks old female wild-type C57BL/6J mice (purchased from Beijing Vital River Laboratory Animal Technology Co., Ltd., China) were randomly divided into three groups (12 mice in each group): the *P.gingivalis* oral infection group (Pg group), the *F.nucleatum* oral infection group (Fn group), and the control group (C group). All mice were maintained at a temperature of 22–25 °C with a 12 h/12 h reverse light/dark cycle and were provided with sterile food and water. The study was approved by the Animal Ethics Committee of Capital Medical University.

### Bacteria preparation

*P. gingivalis* (ATCC 33,277) was cultured in brain heart infusion (BHI) blood agar medium supplemented with hemin (5 µg/mL), Vitamin K1 (1 µg/mL) and 5% defibrinated sheep blood [13, 14]. *F. nucleatum* (ATCC 25,586) was cultured in BHI blood agar medium [15]. Both bacteria were grown under anaerobic conditions with 10% H_2_, 10% CO_2_ and 80% N_2_ at 37 °C. After 5–7 days, single colonies were isolated from the plates. Subsequently, *P. gingivalis* was cultured in BHI broth medium supplemented with the same materials, while *F. nucleatum* was cultured in BHI broth medium under anaerobic conditions at 37 °C for 2 days. The optical density values of the culture medium were determined using a spectrophotometer, and the bacterial concentration was calculated using a pre-prepared standard curve of bacterial concentrations. The bacteria were collected, washed three times with phosphate-buffered saline (PBS), and then resuspended in carboxymethylcellulose (CMC) solution to prepare a 2% CMC solution containing each bacterium at a concentration of 5 × 10^9^ colony-forming units (CFU)/mL.

### Mice oral infection model

**Fig. 1:**
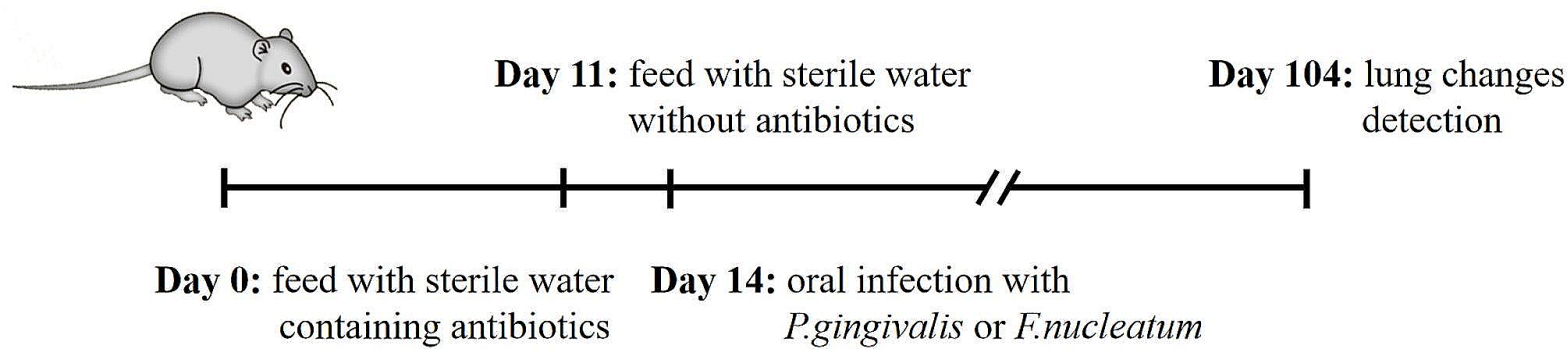
Diagram of the establishment protocol for mouse oral infection models

All mice were fed with sterile water containing trimethoprim (0.16 mg/mL) and sulfamethoxazole (0.8 mg/mL) (Teva Pharmaceutical Industries, China) for 10 days from day 0, then fed with sterile water without antibiotics for 3 days. The mouse model establishment was initiated on day14 [16]. In the Pg group and Fn group, prepared CMC suspension of *P.gingivalis* or *F.nucleatum* (5 × 10^9^ CFU/mL in 2% CMC, 15 µl each time) were smeared around the gums of mice bilateral maxillary second molars separately, and repeated 3 times, once every other day for 3 months. Mice in the C group were applied with a 2% CMC solution without bacteria. Postoperative fasting and water were required for 30 min after application in all groups. The protocol for establishing the mouse model is depicted in Fig. 1.

### Detection of lung function

The FinePointe NAM system (Buxco, USA) was used to noninvasively detect the lung function of the mice. The mice were placed in the testing room with a quiet environment for 1 h, and then the mice were put into the device while they were awake. The lung function indexes were detected according to the instructions of the instrument. After the data had stabilized, the formal test was conducted and the data were recorded. The formal test duration for each mouse was approximately 5 min. The measured lung function indexes included airway resistance (Raw), peak inspiratory/expiratory flow (PIF/PEF), and expiratory flow-50% (EF50). The data for each index were recorded and analyzed using the FinePointe system software.

### Lung tissue samples collection and preservation

After the lung function test, the mice were sacrificed by intraperitoneal injection with an overdose of 5% phenobarbital sodium. Part of the left upper and right anterior lobe of the lung was collected. The tissue was fixed in 10% formalin for histopathology examination, and the remaining lung tissue was used for inflammatory factors and tissue damage associated factors detection by ELISA.

### Measurement of the alveolar bone resorption

The mouse maxilla was boiled in boiling water for 20 min, then the gingival and mucosal tissues on the alveolar bone surface were removed. Following staining with 1% methylene blue for 45 s, the alveolar bone was washed with running water and then dried in the oven at 37 °C. The distance between the cemento-enamel junctions and the alveolar crest of bilateral maxillary molars was measured under a stereomicroscope (10X). Six sites were measured in each maxillary second molar, including mesio-buccal, midbuccal, disto-buccal, mesio-palatal, midpalatal and disto-palatal sites. Each site was measured three times, and the average distance of six sites was calculated as the alveolar bone resorption level of each tooth.

### Histology and immunofluorescence examinations

The fixed lung tissue was dehydrated and then routinely embedded in paraffin. Tissue sections, each 5 μm in thickness, were stained with hematoxylin and eosin (HE) to observe lung tissue structure and inflammatory cell infiltration under a microscope. Immunofluorescence staining was conducted to detect *P. gingivalis* or *F. nucleatum* in the lung tissue samples from each group. The primary antibodies used were anti-*P. gingivalis* (Sigma-Aldrich, USA) (1:400) for *P. gingivalis* detection, and rabbit anti-F. nucleatum (Diatheva, Italy) (1:400) for *F. nucleatum* detection. Subsequently, the sections were incubated with the respective secondary antibodies: AF594 goat anti-rabbit IgG (H + L) (Lablead, China) (1:500) and AF488 goat anti-rabbit IgG (H + L) (Lablead, China) (1:500). Then the sections were observed under a fluorescence microscope (Olympus, Japan).

### ELISA assay

The harvested lung tissues in RIPA buffer with proteinase inhibitor cocktail (Sigma, USA) were homogenized using a Dounce glass homogenizer. The ultrasonic method was used to lyse the cell membrane, and the supernatant was collected and diluted after centrifugation at 5,000 rpm for 15 min for subsequent experiments. Inflammatory factor expression levels of interferon-γ (IFN-γ), tumor necrosis factor (TNF)-α, interleukin (IL)-1β and IL-6 and tissue damage associated factors matrix metalloproteinases-8 (MMP-8) and neutrophil elastase (NE) in the lung tissue homogenate were detected with ELISA kits (PeproTech, USA) following the manufacturer’s instructions. The absorbance values were read at 450 nm by a Synergy HT Microplate Reader (BioTek, USA), and the concentrations were calculated according to the standard curve.

### Statistical analysis

Analysis was performed using the SPSS 19.0 software (SPSS Inc. USA). Data were expressed as mean ± standard deviation (SD) or median (25% quantile, 75% quantile) for normally or nonnormally distributed data, and the independent samples t-test or Mann-whitney U test was used to compare the difference between groups. *P* < 0.05 was considered significantly different.

## Results

### Alveolar bone resorption condition

**Fig. 2:**
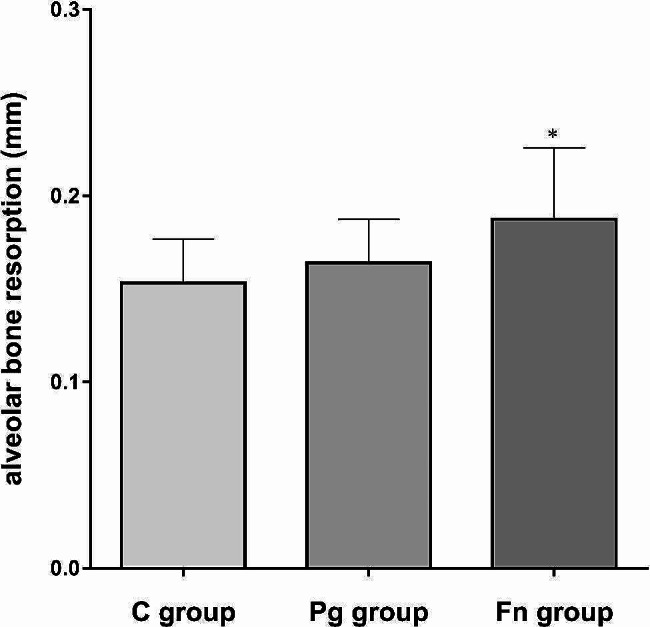
Alveolar bone resorption at the maxillary second molars of mice in each group. The average bone resorption was 0.15 ± 0.02 mm (C group), 0.16 ± 0.02 mm (Pg group) and 0.19 ± 0.04 mm (Fn group) separately (C group: control group, Pg group: *P.gingivalis* oral infection group, Fn group: *F.nucleatum* oral infection group; * vs. C group, *P* < 0.05)

The average alveolar bone resorption of maxillary second molars in each group was 0.15 ± 0.02 mm (C group), 0.16 ± 0.02 mm (Pg group) and 0.19 ± 0.04 mm (Fn group). The Fn group mice showed higher alveolar bone resorption level than the C group (*P* < 0.05). The alveolar bone resorption levels of the Pg group and the C group were not significantly different (*P* > 0.05) (Fig. 2).

### Lung function comparison

**Fig. 3:**
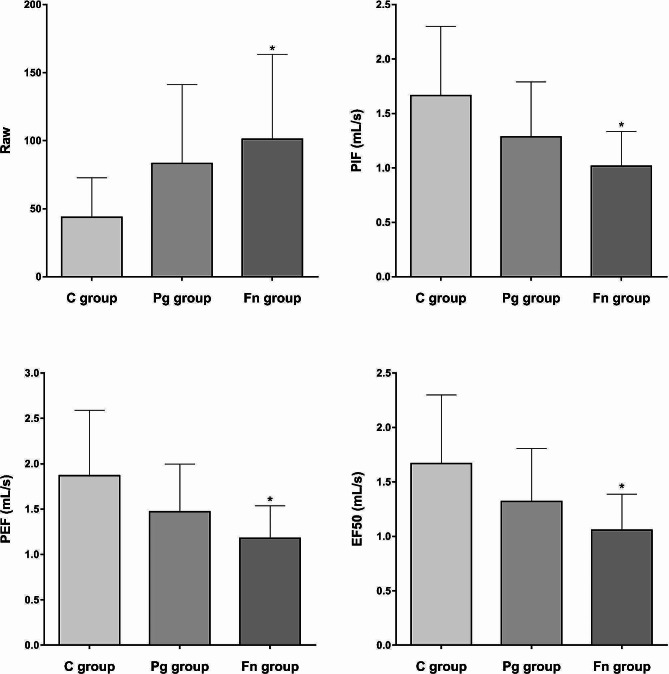
Oral infection with *F.nucleatum* significantly impairs lung function in mice. The level of Raw was significantly increased in the Fn group compared to the C group. The levels of PIF, PEF, and EF50 in the Fn group were significantly decreased compared to the C group. (C group: control group, Pg group: *P.gingivalis* oral infection group, Fn group: *F.nucleatum* oral infection group; Raw: airway resistance, PIF: peak inspiratory flow, PEF: peak expiratory flow, EF50: expiratory flow-50% .* vs C group, *P* < 0.05)

The lung function indexes of each group are shown in Fig. 3. Compared with the C group, the level of Raw was significantly higher in the Fn group (*P* < 0.05). Meanwhile, the levels of PIF, PEF, and EF50 in the Fn group were significantly lower than the C group (all *P* < 0.05). The Pg group showed an increasing trend in the Raw level and decreasing trends in the PIF, PEF, and EF50 levels compared to the C group; however, these differences were not statistically significant (all *P* > 0.05).

### Pulmonary histological manifestations

**Fig. 4:**
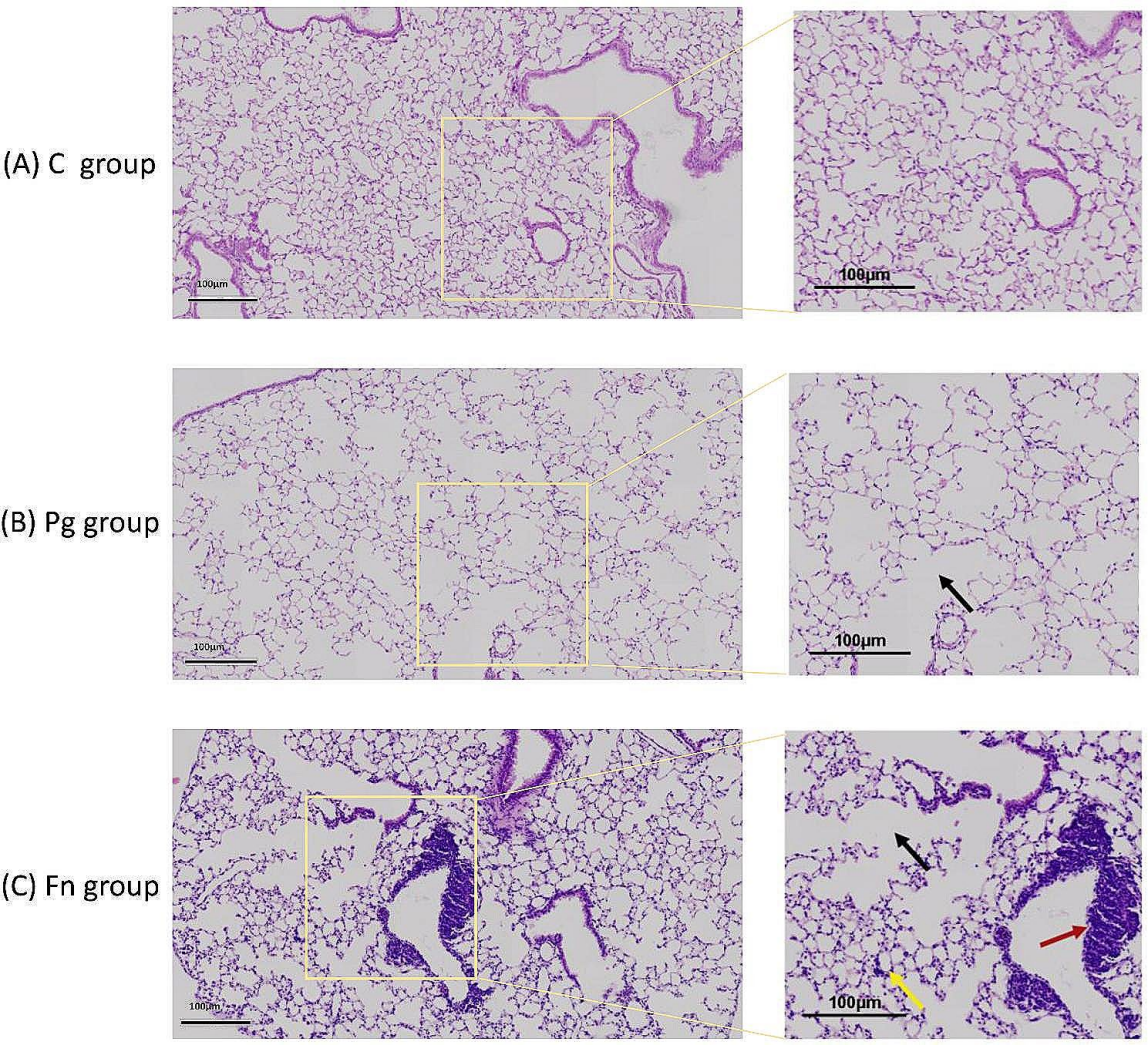
Histological manifestations of the mice lung tissues in each group (HE staining). Oral infections with *P.gingivalis* or *F.nucleatum* led to increased immune cell infiltration, mucous accumulation and airway wall thickening in lung tissue, features characteristic of the advanced stages of COPD(C group: control group, Pg group: *P.gingivalis* oral infection group, Fn group: *F.nucleatum* oral infection group; black arrow: alveolar walls were damaged and fused to form bullae; yellow arrow: inflammatory cells infiltrated around the alveoli; red arrow: the bronchus is infiltrated by a large amount of inflammatory cells)

The lung tissue histological manifestations of the mice in each group are shown in Fig. 4. In the C group, the lung tissue showed normal structure and without obvious inflammatory changes. In contrast, both the Pg group and Fn group showed that the alveolar wall was damaged and fused to form bullae. In the Fn group, inflammatory manifestation was remarkable as a large amount of inflammatory cells distributed around the alveoli and gathered around the bronchus.

### P.gingivalis or F.nucleatum in the lung tissues

**Fig. 5:**
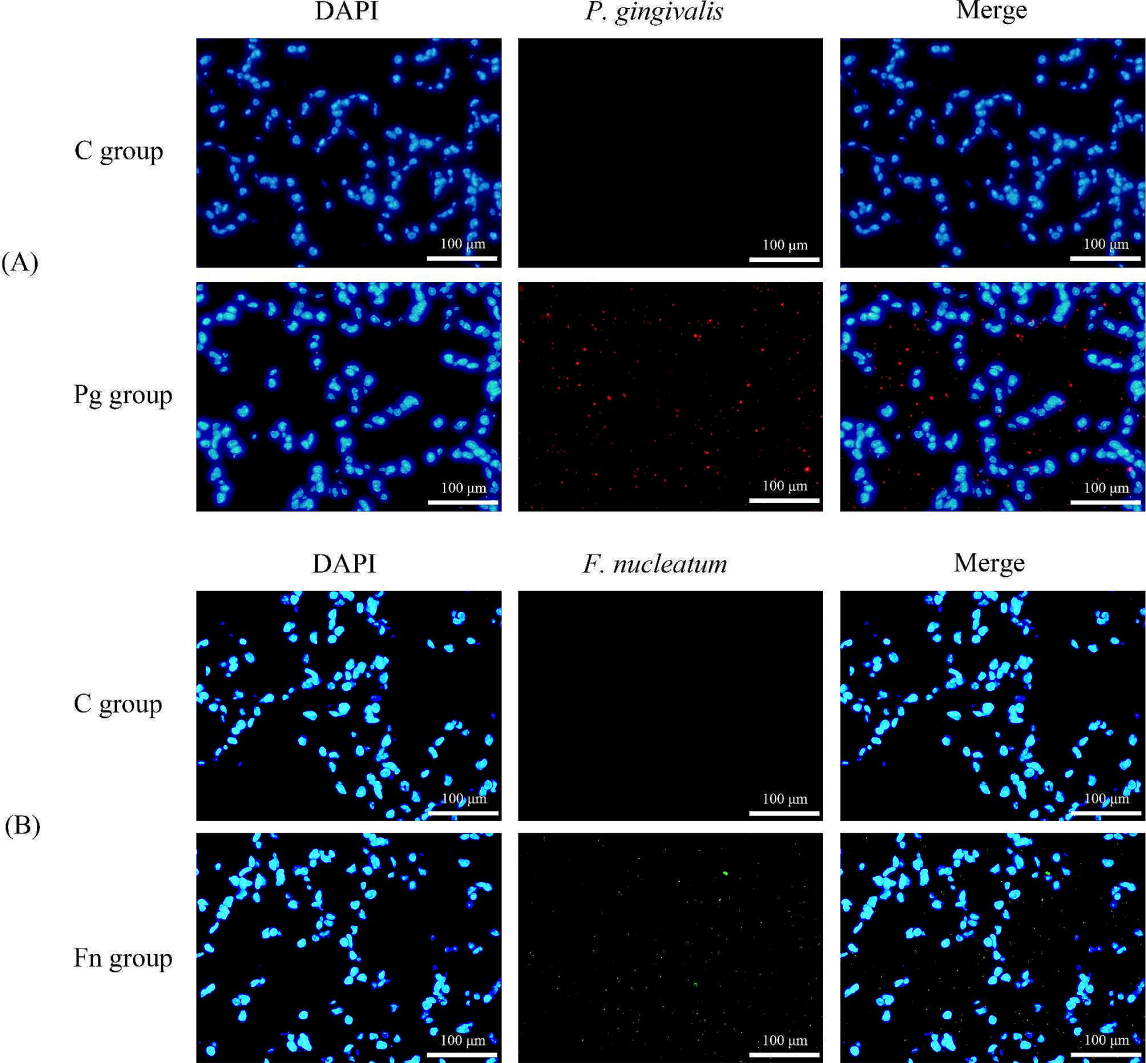
*P. gingivalis* and *F. nucleatum* detection in the lung tissue of each group by immunofluorescence staining. **(A)** positive staining for *P. gingivalis* (red) observed in the Pg group, and no staining was observed in the C group; **(B)** positive staining for *F. nucleatum* (green) observed in the Fn group, and no staining was observed in the C group (C group: control group, Pg group: *P.gingivalis* oral infection group, Fn group: *F.nucleatum* oral infection group)

Immunofluorescence detection revealed positive staining for *P. gingivalis* in the Pg group and for *F. nucleatum* in the Fn group, which suggests the presence of these bacteria in the respective groups. In contrast, no staining was observed for either *P. gingivalis* or *F. nucleatum* in the C group, as depicted in Fig. 5.

### Inflammatory factors expression levels in the lung tissues

**Fig. 6:**
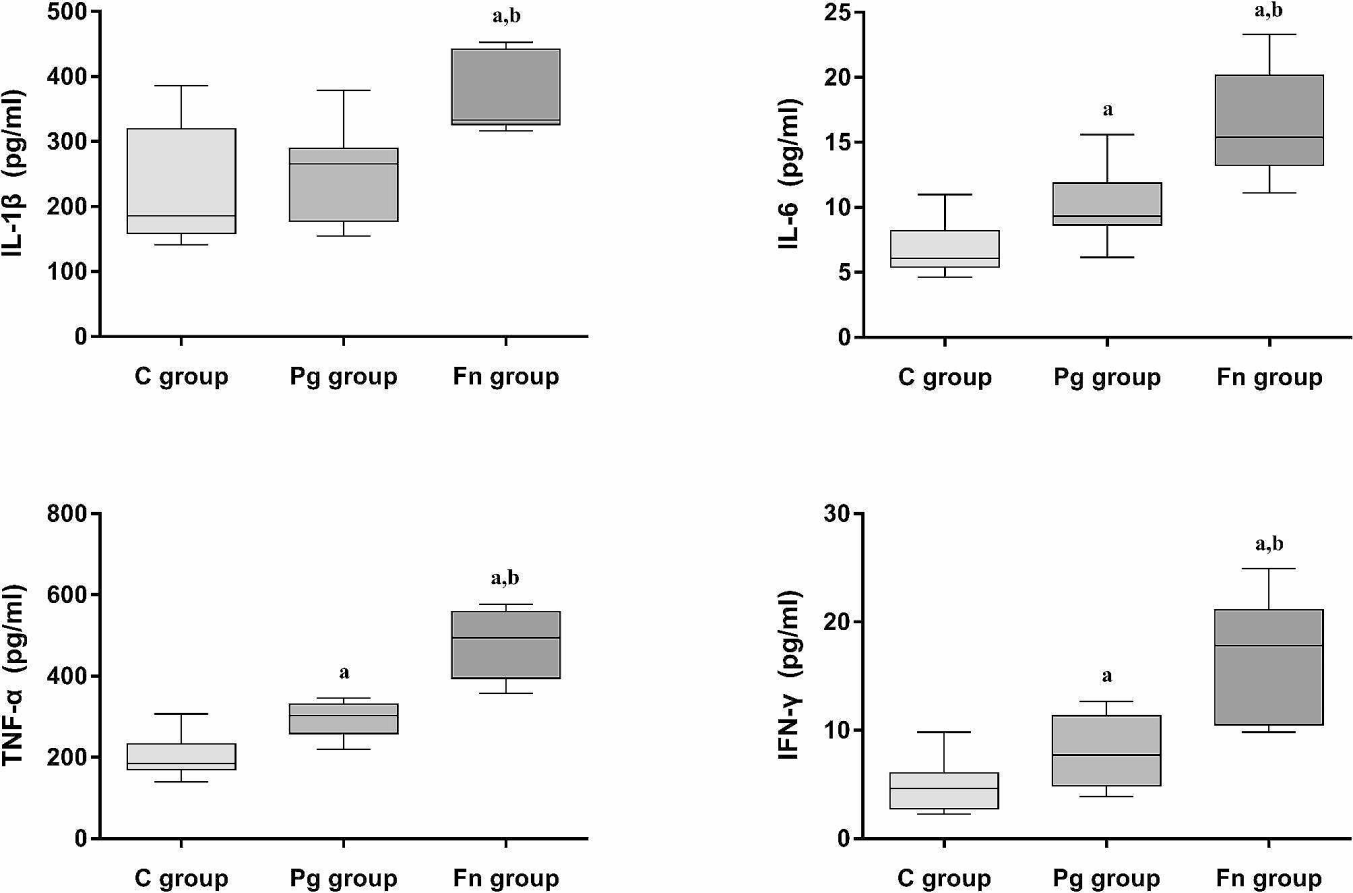
Expression levels of inflammatory factors in the lung tissues of mice in each group. The protein expression level of inflammatory factor IFN-γ, TNF-α and IL-6 were significantly increased both in the Pg group and Fn group, with the levels in the Fn group being significantly higher than those in the Pg group (C group: control group, Pg group: *P.gingivalis* oral infection group, Fn group: *F.nucleatum* oral infection group; a: vs. C group, *P* < 0.05; b: Fn group vs. Pg group, *P* < 0.05)

The expression levels of inflammatory factors in the lung tissues of mice are shown in Fig. 6. In the Pg group, the levels of IL-6, TNF-α and IFN-γ were all significantly elevated compared to the C group (all *P* < 0.05). Similarly, in the Fn group, the levels of IL-1β, IL-6, IFN-γ and TNF-α were also significantly increased compared to the C group (all *P* < 0.05). Furthermore, the levels of IL-1β, IL-6, IFN-γ and TNF-α in the Fn group were higher than those in the Pg group (all *P* < 0.05).

### Tissue damage associated factors expression levels in the lung tissues

**Fig. 7:**
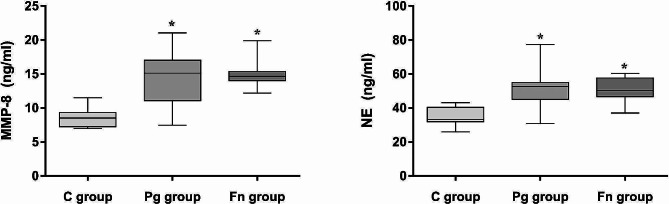
Expression levels of tissue damage associated factors in each group. The tissue damage associated factors NE and MMP-8 in the lung tissues of Pg group and Fn group were both significantly higher than those in the C group (C group: control group, Pg group: *P.gingivalis* oral infection group, Fn group: *F.nucleatum* oral infection group; *vs. C group, *P* < 0.05)

The protein expression levels of MMP-8 and NE in the lung tissues of mice in the Pg group and Fn group were both significantly higher than those in the C group (all *P* < 0.05). However, there was no significant difference in the expression levels between the Pg group and Fn group (*P* > 0.05), as shown in Fig. 7.

## Discussion

In this study, we established an animal model of oral periodontal pathogen infection by inoculating *P. gingivalis* or *F. nucleatum* around the maxillary second molars to investigate their role in the pathogenesis of COPD. The results demonstrated that these bacteria could migrate from the oral cavity to the lung tissue, inducing a decline in lung function, inflammation, and tissue damage, which are similar to the pathological features of COPD. To our knowledge, this is the first study using a mouse model to investigate whether only oral periodontal pathogen infection could directly affect the onset of COPD.

Studies have demonstrated that periodontal pathogens contribute to the pathogenesis of periodontal diseases by selectively modulating the host’s immune-inflammatory response [17]. There are more than 500 species of periodontal bacteria, and *P.gingivalis* and *F.nucleatum* are widely regarded as key pathogens to periodontal disease [18]. Both *P. gingivalis* and *F. nucleatum* are Gram-negative anaerobic bacteria, capable of surviving in hostile environments. In this study, we used *P.gingivalis* and *F.nucleatum* as representative pathogens to investigate the potential role of periodontal pathogens in contributing to COPD pathogenesis.

As our results shown, inoculation with human oral bacteria *P.gingivalis* (Pg group) and *F.nucleatum* (Fn group) around the maxillary second molars both induced alveolar bone resorption of mice. However, only the Fn group exhibited a significant difference compared to the C group. A study has indicated that mixed infection with *P.gingivalis* and *F.nucleatum* induced significant alveolar bone loss whereas neither *P. gingivalis* nor *F. nucleatum* alone did, when compared with non-infected animals [19]. Another study showed that a significant decrease in residual supportive bone volumes was observed in *P.gingivalis* infected groups compared to the control group [20]. Discrepancies in the results may be attributed to differences in the smear dose and duration of exposure. Additionally, minimal alveolar bone resorption was observed in the C group mice, which may be partially due to the CMC solution causing plaque accumulation around the teeth.

COPD is primarily characterized by irreversible airflow obstruction, and the lung function test is the gold standard for its clinical diagnosis [2]. The lung function of COPD patients is characterized as an increase in the degree of airway obstruction [21]. Raw, a measure of airway obstruction, is calculated as the ratio of the mouth-to-alveoli pressure difference to the air flow rate [22]. PIF is the maximal flow achieved during an inspiratory manoeuvre, which is measured by spirometry or using devices designed to mimic inhaler-specific resistance during clinical practice [23]. PEF is the maximum flow that normally occurs at high lung volumes near total lung capacity and is widely measured to assess and monitor airway obstruction [24]. EF50 is the expiratory flow at the mid-tidal volume, which is a useful parameter for phenotypic characterization of lung function and appropriate to monitor bronchoconstriction [25]. The results showed that a significant increase in Raw and decreases in PIF, PEF and EF50 in the Fn group, indicating that the mice exhibited airflow obstruction characteristics similar to those of COPD. The Pg group also showed an airway obstruction trend, although no significant difference was observed.

The pathophysiological process of COPD involves structural remodeling of the epithelial microenvironment and destruction of lung parenchyma, which may lead to a loss of alveolar elastic recoil and airflow restriction. As COPD stages advance, there is an increase in immune cell infiltration, characterized by a predominance of innate immune cells, mucus accumulation, and airway wall thickening [1]. The histological findings of this study partially revealed these similar characteristics. Both the Pg group and Fn group showed damaged alveolar walls and bullae fusion, while the Fn group showed inflammatory changes with numerous inflammatory cells infiltration around the alveoli and aggregation around the bronchus.

Inflammatory factors play important roles in the pathogenesis of COPD [26]. TNF-α, IL-1β and IL-6 were inflammatory mediators produced by epithelial cells when stimulated by inhaled irritants [27]. TNF-α, IL-1β, IL-6, and IFN-γ were also proinflammatory cytokines which may play an important part in many pathobiological processes of COPD [28]. To further explore whether the inflammatory changes in the lung were similar to those in COPD, we examined those inflammatory factors levels in the mice’s lung tissues. The results showed that the protein expression levels of inflammatory cytokines IFN-γ, TNF-α, and IL-6 were significantly increased in the Pg group and Fn group. In addition, the results also showed that the inflammatory factors in the lung tissue of mice in the Fn group were significantly higher than those in the Pg group (*P* < 0.05), indicating that Fn infection has a stronger effect on the pulmonary inflammation. These findings were largely consistent with the histological evidence of lung inflammation in the mice.

Previous study showed that the common pathophysiological process of COPD and periodontal disease includes tissue destruction due to proteolysis of connective tissue proteins by neutrophil proteases (NE) [29], which appear to contribute to the pathogenesis of COPD [30]. NE could promote alveolar cavity enlargement and aggravate the airway inflammation, leading to transient airway stenosis and obstruction, then causing the decrease of ventilation function in the lung [31]. Matrix metalloproteinases (MMPs) are a family of zinc-dependent extracellular matrix remodeling endopeptidases that participate in degradation and remodeling of tissues [32]. A previous study has proved that MMP-8 was increased in the sputum of COPD patients and its expression level was positively correlated with airflow obstruction [33]. The lung tissue MMP-8 and NE protein expression levels in the mice infected with *P.gingivalis* or *F.nucleatum* were significantly increased, which indicated that the periodontal pathogens infection might cause the destruction and remodeling of the lung tissue by increasing the expression of MMP-8 and NE.

In this study, we found that the lungs of mice in the Pg group and Fn group showed COPD-like changes. A possible underlying mechanism of these changes is the migration of *P. gingivalis* and *F. nucleatum* to the lung tissue, which induced subsequent lung inflammation and damage. The immunofluorescence detection results of this study confirmed that *P. gingivalis* or *F. nucleatum had* migrated to the lung from the oral cavity of the mouse model. It is thought that there are two possible pathways for periodontal pathogens to migrate to the lungs: through aspiration or through the circulatory system [34].

In this study, we observed that *F. nucleatum* had a more pronounced destructive effect on lung tissue compared to *P. gingivalis*. These bacteria possess distinct virulence factors: *P. gingivalis* is equipped with proteolytic enzymes, a capsule, lipopolysaccharide, fimbriae, nucleoside diphosphate kinas, ceramide, and outer membrane vesicles, while *F. nucleatum* produces adhesins, endotoxins, and serine proteases [17]. The differential expression and function of these virulence factors may elicit distinct immune responses from the host. For example, F. nucleatum infection more readily triggers the NLRP3 inflammasome, leading to pyroptosis and the release of inflammatory cytokines IL-1β and IL-18, compared to P. gingivalis infection [17]. Further investigation is needed to elucidate the mechanisms by which *F. nucleatum* induces more severe pulmonary changes in mice than *P. gingivalis*.

Previous studies have investigated the effects of periodontal pathogens on pulmonary health in animal models. Feng et al. established a COPD model in rats by combining smoke exposure with intratracheal instillation of Escherichia coli lipopolysaccharide, and then assessed the impact of *P. gingivalis* oral infection on the severity of COPD. Their findings indicated that *P. gingivalis* infection could lead to a further decline in pulmonary function and an aggravation of alveolar inflammation and damage in rats [14]. Xiong et al. established a periodontitis mouse model using tooth ligation combined with P. gingivalis oral infection, and followed by the COPD model establishment by combining smoke exposure with intratracheal injection of porcine pancreatic elastase. The results showed that the periodontitis and COPD could mutually aggravate disease progression [35]. Hayata et al. intratracheally inoculated heat-inactivated periodontal pathogens into mice, revealing that *F. nucleatum* infection could induce higher levels of inflammatory cytokine expression in the lungs compared to *Streptococcus pneumoniae* infection [36]. Tian et al. established a periodontitis model in mice using tooth ligation combined with *P. gingivalis* oral infection, demonstrating that this model caused more severe pulmonary inflammation compared to a model established solely by ligation [37]. Collectively, these studies suggest that periodontal pathogens such as *P. gingivalis* or *F. nucleatum* may play a role in promoting the development of COPD or pulmonary inflammation. However, to our knowledge, no studies have directly investigated whether oral periodontal pathogen infection, excluding other factors, can induce the onset of COPD. This study demonstrates that oral infection with periodontal pathogens alone can induce COPD-like changes in the lungs of mice, further highlighting the potential significant role of periodontal pathogens in the mechanisms linking periodontitis with COPD.

This study has several limitations. First, the research was conducted using only female mice for model establishment, which did not eliminate the potential impact of sex on the study’s outcomes. Second, the study did not specify the pathways by which *P. gingivalis* and *F. nucleatum* migrate to the lungs, such as through aspiration or though the circulatory system. Third, the study did not investigate the potential synergistic effects of simultaneous oral infections with *P. gingivalis* and *F. nucleatum* on the mouse lungs. The underlying mechanism of oral infection with *P. gingivalis* and *F. nucleatum* induced COPD-like changes in the lung of mice is needed to be further studied in the future.

## Conclusion

In this study, three months after the oral infection with *P. gingivalis* or *F. nucleatum*, these bacteria were found within the lungs of the mice. This migration led to a decrease in lung function, an increase in inflammatory factors and tissue damage associated factors, and pathological changes in lung tissue, including alveolar fusion and inflammation, which are similar to the characteristics of COPD. Moreover, *F. nucleatum* exhibited a more pronounced destructive impact on lung tissue compared to *P. gingivalis*. These findings suggest that the migration of periodontal pathogens to the lungs may contribute to the pathogenesis of COPD and play a significant role in the association between periodontitis and COPD. This may provide potential targets for the prevention and treatment of COPD through periodontal intervention.

## Data Availability

The datasets used and/or analysed during the current study available from the corresponding author on reasonable request.

## Abbreviations

COPD: Chronic obstructive pulmonary disease
Pg/ P.gingivalis: Porphyromonas gingivalis
F.nucleatum: Fusobacterium nucleatum
HE: Hematoxylin and eosin
MMP-8: Matrix metalloproteinases-8
NE: Neutrophil elastase
